# Modeling the Composites for Magnetoelectric Microwave Devices

**DOI:** 10.3390/s23041780

**Published:** 2023-02-05

**Authors:** Mirza Bichurin, Oleg Sokolov, Sergey Ivanov, Elena Ivasheva, Viktor Leontiev, Vyacheslav Lobekin, Gennady Semenov

**Affiliations:** Yaroslav-the-Wise Novgorod State University, 173003 Velikiy Novgorod, Russia

**Keywords:** ferromagnetic metal, piezoelectric, magnetoelectric effect, electromechanical resonance, magnetoacoustic resonance, substrate effect, mismatch effect

## Abstract

Many studies of the ME effect have been carried out in the microwave range in connection with the possibility of creating new electronic devices. One of the main microwave ME effects is the FMR line shift in an electric field, and the purpose of this article is to compare the FMR line shift in the ME structure in an electric field for a number of ferromagnetic metals, their alloys, and YIG ferrite using various piezoelectrics. This article discusses the regimes when the bias field is directed along the main axes of the magnetic component, while, as is known, the observed effect is due only to deformation. As a result of the study, ME structures with maximum and minimum microwave ME effects were found. In addition, the “substrate effect” in the piezoelectric YIG-GGG structure is considered.

## 1. Introduction

Many studies [1,2,3] have been devoted to the research of magnetoelectric (ME) interaction in the microwave range, which is associated with the possibility of developing new electronic devices. The first studies were related to the ferromagnetic resonance (FMR) line shift due to elastic deformation under the action of an external electric field on the ME composite. This is the so-called inverse ME effect. At the same time, the problems of using bulk and polycrystalline ME composites with a broad FMR line for this purpose were revealed, and the need to use a single-crystal magnetic component with a narrow FMR line in a layered composite was found out [4,5,6]. Along with these works, studies of hybrid spin-electromagnetic waves in ferrite-ferroelectric layered structures were carried out without the participation of interface elastic interaction. The results of such an investigation were presented in [7]. Besides, a new ME effect was discovered when the frequencies of electromechanical resonance (EMR) in the piezoelectric phase and FMR in the magnetic phase coincided, the so-called ME effect in the region of magnetoacoustic resonance (MAR) [8]. In this case, a sharp increase in the ME effect is expected.

All these studies were carried out, as a rule, with the bias field oriented along the crystallographic axes. Subsequent works, in which the angular dependences of the ME effect were studied, showed that for a number of orientations of the bias field, the ME effect is much larger compared with the effect measured along the principal axes [9]. The reasons for this can be related to both two-magnon scattering [10,11] and the accumulation of charges at the interface [12]. An increase in the ME effect when the bias field is oriented along directions different from the main ones gives hope for practical application, although the FMR line-width also increases in this case [13].

In terms of practical applications, the first reports on the ME microwave devices, such as phase shifters and attenuators, were made at the International Conferences, MEIPIC-3 in 1996 [14] and MEIPIC-4 in 2001 [15], respectively. Furthermore, the characteristics of ME microwave devices were improved, and the range of proposed devices was expanded significantly. In recent years, many publications have appeared on the development of devices such as ME bandpass filters [16,17,18,19,20], ME phase shifters [21,22,23], ME isolators [24,25,26], and ME antennas [27,28,29,30,31].

The main purpose of this article is practical. The authors considered the issues that researchers of the microwave ME effect face: how to choose a ferromagnetic metal (FM) and a piezoelectric as components of the ME composite to obtain the maximum ME effect, and how the substrate affects its value. The authors provided answers to these questions using specific examples. It should be noted that the results obtained in the evaluation of the “substrate effect” for YIG-GGG are original.

This article is organized as follows. First, in Section 2 and Section 3, we discuss the FMR line shift in an electric field in composites based on FM and yttrium iron garnet (YIG) with various piezoelectrics. Specific examples reveal the result for the orientations of the bias field along crystallographic axes, i.e., for the strain-mediated ME effect. Furthermore, in Section 4, the “substrate effect” due to the presence of a layer of gadolinium gallium garnet (GGG) in the structure of the piezoelectrics-YIG-GGG is considered.

## 2. ME Effect in Ferromagnetic Metal—Piezoelectric

Studies of the microwave ME effect in nanostructures (10–100 nm) based on FM and their alloys are of great practical interest since the transition to the nanoscale often leads to the appearance of new properties of nanofilms compared with single crystals, which can be used in the new field of technology, in particular, in spintronics [32]. The research interest in FM nanofilms can be explained by the following factors. The technology for obtaining such structures based on magnetron sputtering is simple and affordable compared with ferrite technology, although it should be noted that the production of a single-crystal FM film with a narrow FMR line is a complex technological task. In addition, the amplitude of the FMR line of such a nanofilm significantly exceeds the amplitude of the line of a ferrite nanofilm, making it possible to design nanodevices using standard microwave measuring techniques. Therefore, we decided to consider the microwave ME effect in nanostructures based on Ni, Fe, Co, and their alloys and various piezoelectrics in a separate section.

**Figure 1:**
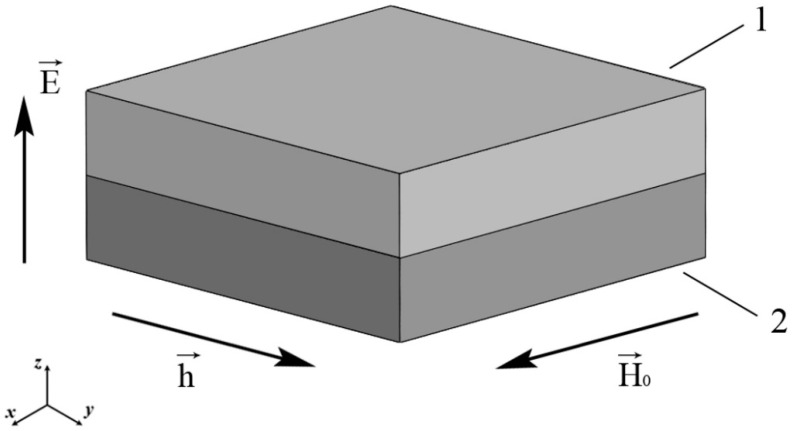
Two-layer ME structure.

Consider the two-layer ME structure consisting of magnetic and piezoelectric phases in Figure 1.

In Figure 1, a ME structure consisting of 1 (magnet) and 2 (piezoelectric) with the directions of the bias and alternating magnetic fields and the external electric one is shown. We will consider an FM as a magnet. The sample is oriented so that the piezoelectric thickness corresponds to the crystallographic direction [011] in the case of lead magnesium niobate-lead titanate (PMN-PT) and lead zinc niobate-lead titanate (PZN-PT). Furthermore, in the case of PMN-PT, the directions of the x and y axes coincide with the crystallographic directions [100] and [01−1], respectively.

Let us consider a thin nickel film to which a bias field is applied along the film plane. We will assume that the value of this field is sufficiently large and the nickel film is uniformly magnetized to saturation. Axis 1 (x) is directed along *H_0_*, then it has components (H_0_, 0, 0), and the equilibrium magnetization has components (M_0_, 0, 0). In this case, the 1 (x) axis is directed along the easy magnetization axis of the nickel film.

The Landau–Lifshitz–Gilbert equation [32] in a thin nickel film under the action of a high-frequency magnetic field in the presence of a bias field *H*_0_ under the condition *h* << *H*_0_ and considering the dissipation:

(1) $$\frac{\partial \vec{M}}{\partial t}=\gamma \left[\vec{M}\times \frac{\partial W}{\mu_{0}\partial \vec{M}}\right]+i\omega \alpha \frac{\left[{\vec{M}}_{0}\times \vec{m}\right]}{M_{0}}$$

where the total magnetization is the sum of the equilibrium ${\vec{M}}_{0}$ and high frequency $\vec{m}$ constituents:

(2) $$\vec{M}={\vec{M}}_{0}+\vec{m}$$

where free energy density:

(3) $$W=-\mu_{0}\vec{M}\cdot \vec{H}+\frac{\mu_{0}}{2}\sum N_{11}^{i}M_{1}^{2}+\frac{\mu_{0}}{2}\sum N_{22}^{i}M_{2}^{2}+\frac{\mu_{0}}{2}\sum N_{33}^{i}M_{3}^{2}$$

where *γ* is a gyromagnetic ratio, *μ_0_* is a magnetic constant, *ω* is a cyclic oscillation frequency, and *α* is a dissipation parameter.

Equation (1) is valid if there are demagnetizing factors $N_{kk}^{i}$ of any nature in the sums in Equation (3). However, since before the beginning of the next section, we will consider FMR in FM and their alloys without taking into account the influence of the presence of a piezoelectric, we will have in mind the summation only over demagnetizing factors associated with the shape and magnetic uniaxial anisotropy. For the sake of brevity in writing formulas, we will simply use the sum sign.

To solve (1), using the method of linearization in small parameters, we obtain a linearly inhomogeneous system of 3 equations with 3 unknowns. Solving it by the Cramer method, we obtain a high-frequency magnetic susceptibility tensor, which connects the components of the high-frequency component of magnetization with the components of the high-frequency component of the magnetic field. The imaginary parts of the complex components of the high-frequency magnetic susceptibility tensor ${\chi^{\prime \prime }}_{22},\;{\chi^{\prime \prime }}_{33}$ were found:

(4) $$\begin{array}{l} {\chi^{\prime \prime }}_{22}=-\alpha \gamma M_{0}\omega \frac{\omega^{2}\left(1+\alpha^{2}\right)-\omega_{0}^{2}+\gamma^{2}\left(H_{0}+\left\{\sum N_{33}^{i}-\sum N_{11}^{i}\right\}M_{0}\right)\left(2H_{0}+\left\{\sum N_{22}^{i}+\sum N_{33}^{i}-2\sum N_{11}^{i}\right\}M_{0}\right)}{{\left[\omega^{2}\left(1+\alpha^{2}\right)-\omega_{0}^{2}\right]}^{2}+\gamma^{2}\alpha^{2}\omega^{2}{\left(2H_{0}+\left\{\sum N_{22}^{i}+\sum N_{33}^{i}-2\sum N_{11}^{i}\right\}M_{0}\right)}^{2}}\\ {\chi^{\prime \prime }}_{33}=-\alpha \gamma M_{0}\omega \frac{\omega^{2}\left(1+\alpha^{2}\right)-\omega_{0}^{2}+\gamma^{2}\left(H_{0}+\left\{\sum N_{22}^{i}-\sum N_{11}^{i}\right\}M_{0}\right)\left(2H_{0}+\left\{\sum N_{22}^{i}+\sum N_{33}^{i}-2\sum N_{11}^{i}\right\}M_{0}\right)}{{\left[\omega^{2}\left(1+\alpha^{2}\right)-\omega_{0}^{2}\right]}^{2}+\gamma^{2}\alpha^{2}\omega^{2}{\left(2H_{0}+\left\{\sum N_{22}^{i}+\sum N_{33}^{i}-2\sum N_{11}^{i}\right\}M_{0}\right)}^{2}} \end{array}$$

where:

(5) $$\omega_{0}^{2}=\gamma^{2}\left(H_{0}+\left\{\sum N_{22}^{i}-\sum N_{11}^{i}\right\}M_{0}\right)\left(H_{0}+\left\{\sum N_{33}^{i}-\sum N_{11}^{i}\right\}M_{0}\right)$$

Since the 3 (z) axis is directed perpendicular to the film plane, the demagnetizing factors associated with the shape of the sample:

(6) $$\begin{array}{l} N_{11}^{F}=N_{22}^{F}=0\\ N_{33}^{F}=1 \end{array}$$

Since the easy magnetization axis of the nickel film is directed along the 1 (x) axis, the demagnetizing factors associated with the magnetic uniaxial anisotropy are equal to:

(7) $$\begin{array}{l} N_{11}^{a}=-\frac{H_{a}}{M_{0}}\\ N_{22}^{a}=N_{33}^{a}=0 \end{array}$$

The resonant values of the bias field H_0_ for ${\chi^{\prime \prime }}_{22},\;{\chi^{\prime \prime }}_{33}$, which were numerically found in the Maple program for the frequency f = 10 GHz, coincide with each other and with the value found from the resonance condition to within 1 A/m:

(8) $$\omega^{2}=\omega_{0}^{2}$$

Using Equation (5), we can represent Equation (8) as a quadratic one with respect to *H*_0_:

(9) $$\gamma^{2}H_{0}^{2}+\gamma^{2}M_{0}\left(\sum N_{22}^{i}+\sum N_{33}^{i}-2\sum N_{11}^{i}\right)H_{0}-\omega^{2}=0$$

Since the value of *H*_0_ must be positive, then from the 2 roots of the quadratic equation we choose the one with a plus sign in front of the root of the discriminant:

(10) $$H_{0}=\frac{\gamma M_{0}\left(2\sum N_{11}^{i}-\sum N_{22}^{i}-\sum N_{33}^{i}\right)+\sqrt{\gamma^{2}M_{0}^{2}{\left(\sum N_{22}^{i}-\sum N_{33}^{i}\right)}^{2}+4\omega^{2}}}{2\gamma }$$

Substituting (6) and (7) into (10), we obtain $H_{0}=\;148696\;\frac{A}{m}\approx \;1869\;Oe$ for the resonant frequency *f* = 10 GHz, note, that *ω = 2πf*. Similarly, we find the resonant value of the bias field *H*_0_ for other FM and alloys for resonant frequency *f* = 10 GHz:

Fe: $H_{0}=212551\;\frac{A}{m}\approx 2671\;Oe$;

Co: $H_{0}=56628\;\frac{A}{m}\approx 712\;Oe$;

NiFe: $H_{0}=63011\;\frac{A}{m}\approx 792\;Oe$;

FeGaB: $H_{0}=66517\;\frac{A}{m}\approx 836\;Oe$.

When calculating the resonant value of the bias field for the indicated FM and alloys, the material parameters given in Table 1 were used.

### FMR Line Shift in a Two-Layer ME Structure of Ferromagnetic Metal/Piezoelectric

Let us find the demagnetizing factors for the Ni / Piezoelectric structure associated with the action of a constant electric field. Let us consider the case when the constant electric field is directed along the 3 (z) axis.

Consider the mechanical balance of the structure. The boundary conditions for a free sample in the form of a rectangular parallelepiped, the base of which is a square, are equal to:

(11) Tm3=Tp3=0

(12) tmTm1+tpTp1=0,tmTm2+tpTp2=0.

where *^m^T_j_* are the components of the stress tensor of the magnetostrictive phase, *^p^T_j_* are the components of the stress tensor of the piezoelectric phase, *^m^t* is a thickness of the magnetostrictive phase, and *^p^t* is a thickness of the piezoelectric phase.

Considering (7), the constitutive equations for nickel look like:

(13) S1=sm11Tm1+sm12Tm2,S2=sm12Tm1+sm11Tm2

and for piezoelectric:

(14) S1=sp11Tp1+sp12Tp2+d31E,S2=sp12Tp1+sp22Tp2+d32E.

where *S_i_* are the strain tensor components of the magnetostrictive and piezoelectric phases, *^m^s_ij_* are the compliance coefficients of the magnetostrictive phase, *^p^s_ij_* are the compliance coefficients of the piezoelectric phase, and *d_ki_* are the piezoelectric modules.

Solving (12), (13), and (14) together, we find:

(15) Tm1=(d31sm11tp+d31sp22tm−d32sm12tp−d32sp12tm)tpEsm112tp2+sm11sp11tmtp+sm11sp22tmtp−sm122tp2−2sm12sp12tmtp+sp11sp22tm2−sp122tm2Tm2=(d32sm11tp+d32sp11tm−d31sm12tp−d31sp12tm)tpEsm112tp2+sm11sp11tmtp+sm11sp22tmtp−sm122tp2−2sm12sp12tmtp+sp11sp22tm2−sp122tm2

Part of the free energy of nickel associated with the presence of stresses arising from the action of an electric field on a piezoelectric is:

(16) ΔWME=−3λ1002M02(Tm1M12+Tm2M22+Tm3M32)

where *λ_100_* is the magnetostriction coefficient.

Let us write the part of the free energy of nickel associated with the presence of stresses arising from the action of an electric field on a piezoelectric as the corresponding term in Equation (3):

(17) $$\Delta W_{ME}=\frac{\mu_{0}}{2}\sum N_{11}^{E}M_{1}^{2}+\frac{\mu_{0}}{2}\sum N_{22}^{E}M_{2}^{2}+\frac{\mu_{0}}{2}\sum N_{33}^{E}M_{3}^{2}$$

Furthermore, comparing Equations (16) and (17), we find the effective demagnetizing factors arising from the action of the electric field *E*:

(18) N11E=−3λ100μ0M02Tm1, N22E=−3λ100μ0M02Tm2, N33E=0.

We define the resonance condition without considering the effective demagnetizing factors arising from the action of the electric field *E*. Hereinafter, in the signs of the sums over the demagnetizing factors, we indicate that the summation is carried out over all their different origins, except for the action of the electric field:

(19) $$\omega^{2}=\gamma^{2}\left(H_{0}+\left\{\sum_{i\neq E}N_{22}^{i}-\sum_{i\neq E}N_{11}^{i}\right\}M_{0}\right)\left(H_{0}+\left\{\sum_{i\neq E}N_{33}^{i}-\sum_{i\neq E}N_{11}^{i}\right\}M_{0}\right)$$

Next, we write the resonance condition, taking into account the small correction associated with small effective demagnetizing factors arising from the action of the electric field *E*:

(20) $$\omega^{2}=\gamma^{2}\left(H_{0}+\delta H_{E}+\left\{\sum_{i\neq E}N_{22}^{i}-\sum_{i\neq E}N_{11}^{i}+N_{22}^{E}-N_{11}^{E}\right\}M_{0}\right)\left(H_{0}+\delta H_{E}+\left\{\sum_{i\neq E}N_{33}^{i}-\sum_{i\neq E}N_{11}^{i}+N_{33}^{E}-N_{11}^{E}\right\}M_{0}\right)$$

Let us subtract (19) from (20), retaining in the resulting equation only terms of the first order of smallness in $\delta H_{E},N_{11}^{E},N_{22}^{E},N_{33}^{E}$:

(21) $$M_{0}Q_{2}\left(N_{22}^{E}-N_{11}^{E}\right)+M_{0}Q_{3}\left(N_{33}^{E}-N_{11}^{E}\right)+Q_{1}\delta H_{E}=0$$

where:

(22) $$\begin{array}{l} Q_{1}=2H_{0}+\left\{\sum_{i\neq E}N_{22}^{i}+\sum_{i\neq E}N_{33}^{i}-2\sum_{i\neq E}N_{11}^{i}\right\}M_{0}\\ Q_{2}=H_{0}+\left\{\sum_{i\neq E}N_{33}^{i}-\sum_{i\neq E}N_{11}^{i}\right\}M_{0}\\ Q_{3}=H_{0}+\left\{\sum_{i\neq E}N_{22}^{i}-\sum_{i\neq E}N_{11}^{i}\right\}M_{0} \end{array}$$

Now we find the resonance line shift, as:

(23) $$\delta H_{E}=\frac{M_{0}\left[Q_{2}\left(N_{11}^{E}-N_{22}^{E}\right)+Q_{3}\left(N_{11}^{E}-N_{33}^{E}\right)\right]}{Q_{1}}$$

Substitute (6) and (7) into (22):

(24) $$\begin{array}{l} Q_{1}=2H_{0}+M_{0}+2H_{a}\\ Q_{2}=H_{0}+M_{0}+H_{a}\\ Q_{3}=H_{0}+H_{a} \end{array}$$

Then, from (23) we find:

(25) δHE=−3λ100(Q2{Tm1−Tm2}+Q3Tm1)μ0M0Q1

**Figure 2:**
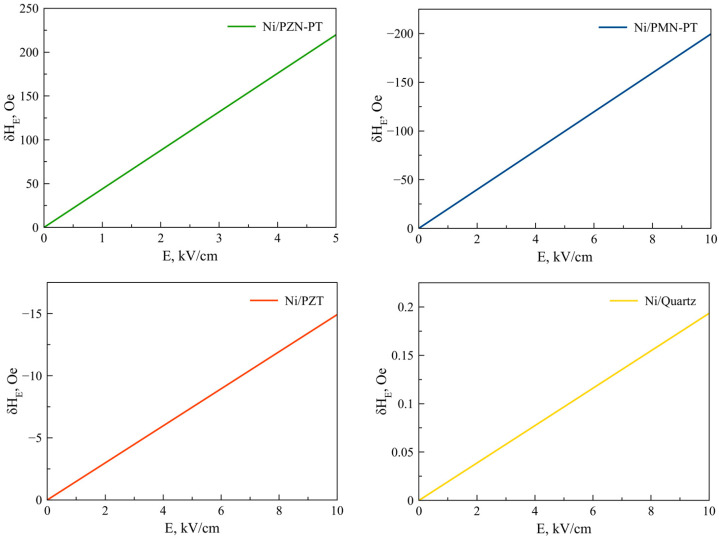
FMR line shift as a function of electric field for Ni/PZN-PT, Ni/PMN-PT, Ni/PZT, Ni/Quartz structures.

**Figure 3:**
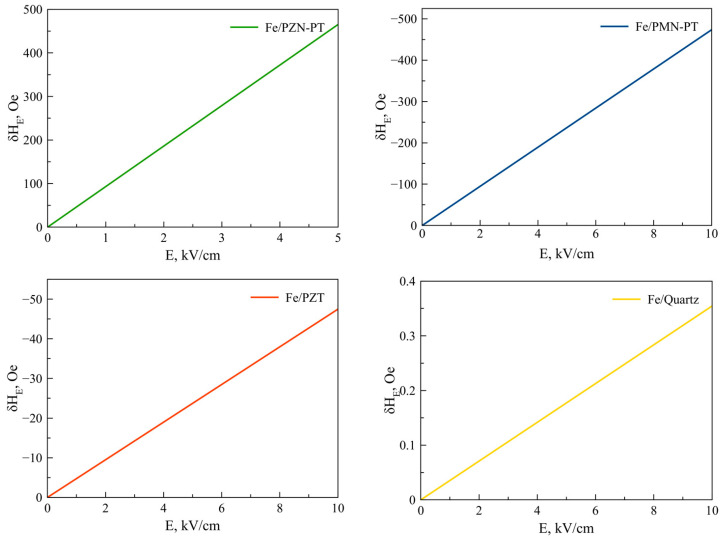
FMR line shifts as a function of electric field for Fe/PZN-PT, Fe/PMN-PT, Fe/PZT, Fe/Quartz structures.

**Figure 4:**
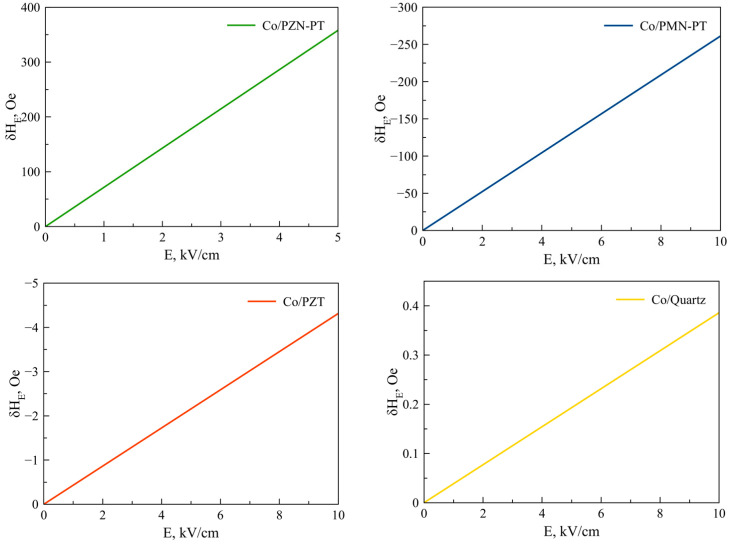
FMR line shifts as a function of electric field for Co/PZN-PT, Co/PMN-PT, Co/PZT, Co/Quartz structures.

**Figure 5:**
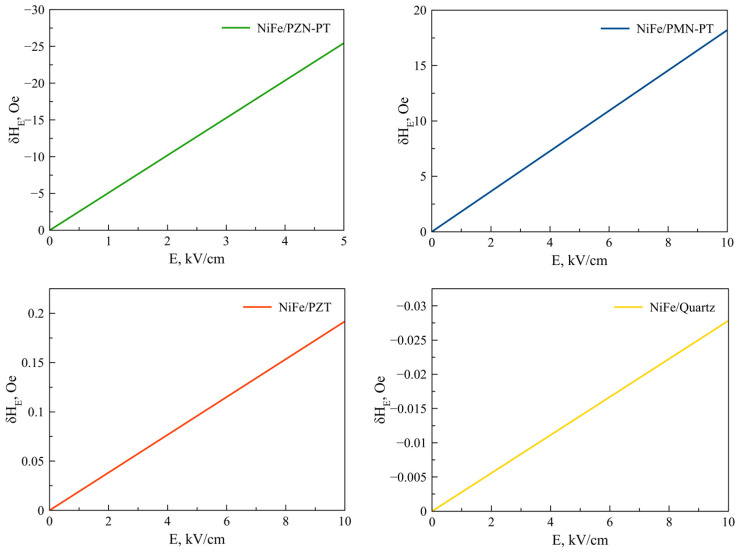
FMR line shifts as a function of electric field for NiFe/PZN-PT, NiFe/PMN-PT, NiFe/PZT, NiFe/Quartz structures.

**Figure 6:**
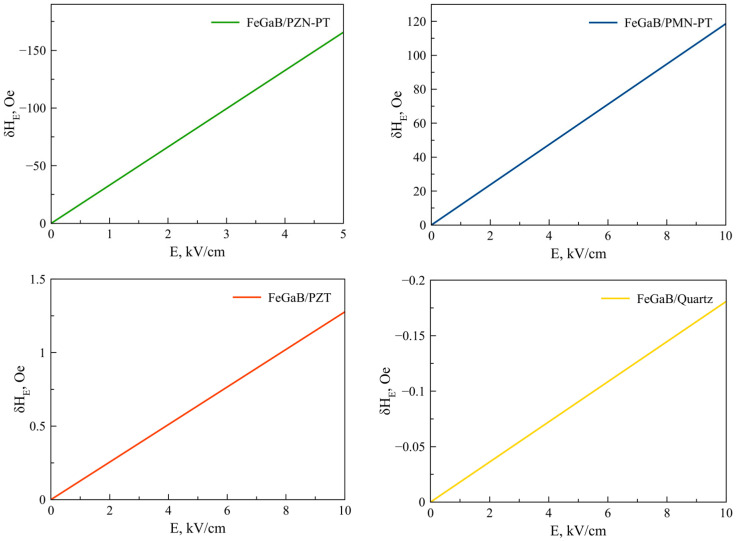
FMR line shifts as a function of electric field for FeGaB / PZN-PT, FeGaB / PMN-PT, FeGaB/PZT, FeGaB/Quartz structures.

Equation (25) is also valid for Fe, Co, NiFe, FeGaB / Piezoelectric structures. Figure 2, Figure 3, Figure 4, Figure 5 and Figure 6 show the dependencies of the FMR line shift on the electric field for ME structures with FM and alloys of Ni, Fe, Co, NiFe, and FeGaB and piezoelectrics of PZN-PT, PMN-PT, lead zirconate titanate (PZT), and Quartz. In this case, the bias field was directed along axis 1 (x).

The material parameters that were used in the calculations are given in Table 1 and Table 2. The thickness of the magnetostrictive phase is *^m^t =* 5 × 10^−8^ m and the piezoelectric one is *^p^t =* 5 × 10^−4^ m.

As can be seen from Equation (25), the FMR line shift depends rather intricately on the corresponding components of the magnetostrictive phase stress tensor *^m^T_1_*, *^m^T_2_*, which, in turn, also depend not quite trivially on the corresponding components of the piezoelectric tensor *d_31_*, *d_32_*. Thus, the sign of the resonant line shift, depending on the total action of all the above factors, can be either positive or negative. As can be seen from the graphs in Figure 2, Figure 3, Figure 4, Figure 5 and Figure 6, the largest FMR line shift is observed in the structure with PZN-PT, and the smallest one is observed in the structure with the Quartz X-cut, which is related to the magnitude of the piezocoefficients. The results obtained show that in order to clearly observe the FMR line shifts in an electric field, it is necessary to have narrow FMR lines for magnetic phases of ME composite.

## 3. ME Effect in Ferrite—Piezoelectric

In ref. [33], a method was developed for calculating the microwave ME effect in two-layer ME structures in which YIG ferrite was used as the magnetostrictive phase (Figure 1). 

Here we present this method of calculation with examples, supplementing the picture with several more piezoelectrics for completeness, and making appropriate conclusions and recommendations for choosing piezoelectrics for observing the maximum microwave ME effect.

Let us consider a thin YIG plate (001), to which a bias field *H_0_* is applied along the plane of the plate. We will assume that the value of this field is sufficiently large and the YIG plate is uniformly magnetized to saturation. Axis 2 (y) is directed along *H_0_*, then it has components (0, H_0_, 0), and the equilibrium magnetization has components (0, M_0_, 0). Axis 1 (x) is directed along the edge of the YIG plate, which coincides with the crystallographic direction [100]. The motion equation of magnetization in a thin YIG plate under the action of h in the presence of *H_0_* under the condition *h* << *H*_0_, considering the dissipation, is given in the previous section. Furthermore, we consider the case when the bias field is directed perpendicular to the plane of the YIG plate. Axis 3 (z) is directed along *H*_0_, then it has components (0, 0, H_0_), and the equilibrium magnetization has components (0, 0, M_0_). Using the equation of motion of the magnetization in the YIG layer, the necessary imaginary parts of the complex components of the high-frequency magnetic susceptibility ${\chi^{\prime \prime }}_{11},\;{\chi^{\prime \prime }}_{33}$ were found.

Free energy density related to magnetic crystalline cubic anisotropy:

(26) $$W_{K}=\frac{K_{1}}{M_{0}^{4}}\left(M_{x}^{2}M_{y}^{2}+M_{y}^{2}M_{z}^{2}+M_{z}^{2}M_{x}^{2}\right)$$

Demagnetizing factors related to sample shape:

(27) $$\begin{array}{l} N_{11}^{F}=N_{22}^{F}=0\\ N_{33}^{F}=1 \end{array}$$

Demagnetizing factors associated with magnetic crystalline cubic anisotropy:

(28) $$N_{11}^{a}=N_{33}^{a}=-\frac{H_{a}}{M_{0}},\;N_{22}^{a}=0$$

The resonant values of the bias field *H*_0_ for ${\chi^{\prime \prime }}_{11},\;{\chi^{\prime \prime }}_{33}$, which were numerically found in the Maple program for the frequency *f* = 10 GHz, coincide with each other with an accuracy of 1 A/m and with the value found from the resonance condition *ω^2^* = *ω_0_^2^*, where:

(29) $$\omega_{0}^{2}=\gamma^{2}\left(H_{0}+\left\{\sum N_{11}^{i}-\sum N_{22}^{i}\right\}M_{0}\right)\left(H_{0}+\left\{\sum N_{33}^{i}-\sum N_{22}^{i}\right\}M_{0}\right)$$

Equation (29) is equivalent in a general sense to equation (19). However, since in Section 2, the bias field is directed along the 1 (x) axis, and in this section the bias field was directed along the 2 (y) axis, Equations (19) and (29) differ by the corresponding permutation of indices 11 and 22.

Then, the expression for calculating the value of the bias field when oriented along the axis 2 (y) has the form:

(30) $$H_{0}=\frac{\gamma M_{0}\left(2\sum N_{22}^{i}-\sum N_{11}^{i}-\sum N_{33}^{i}\right)+\sqrt{\gamma^{2}M_{0}^{2}{\left(\sum N_{11}^{i}-\sum N_{33}^{i}\right)}^{2}+4\omega^{2}}}{2\gamma }$$

Substituting Equations (27) and (28) into Equation (29), we obtain $H_{0}=218501\frac{A}{m}\approx 2746\;Oe$ for the resonant frequency *f* = 10 GHz.

The expression for calculating the value of the bias field when oriented along the axis 3 (z) has the form:

(31) $$H_{0}=\frac{\gamma M_{0}\left(2\sum N_{33}^{i}-\sum N_{11}^{i}-\sum N_{22}^{i}\right)+\sqrt{\gamma^{2}M_{0}^{2}{\left(\sum N_{11}^{i}-\sum N_{22}^{i}\right)}^{2}+4\omega^{2}}}{2\gamma }$$

From Equation (31) we obtain $H_{0}=429649\frac{A}{m}\approx 5399\;Oe$ for the resonant frequency *f* = 10 GHz.

### FMR Line Shift in a Two-Layer Magnetoelectric Structure of YIG/Piezoelectric

The effective demagnetization factors for the YIG / Piezoelectric structure, associated with the action of a constant electric field that is directed along the 3 (z) axis, are similar to the demagnetization factors given in the previous section.

We write the resonance condition without considering the effective demagnetizing factors arising from the action of the electric field E, for the case when the bias field *H_0_* is directed along the axis 2 (y):

(32) $$\omega^{2}=\gamma^{2}\left(H_{0}+\left\{\sum_{i\neq E}N_{11}^{i}-\sum_{i\neq E}N_{22}^{i}\right\}M_{0}\right)\left(H_{0}+\left\{\sum_{i\neq E}N_{33}^{i}-\sum_{i\neq E}N_{22}^{i}\right\}M_{0}\right)$$

Next, we write the resonance condition, taking into account the small correction *^1^δH_E_* associated with small effective demagnetizing factors arising from the action of the electric field *E*:

(33) ω2=γ2(H0+δ1HE+{∑i≠EN11i−∑i≠EN22i+N11E−N22E}M0)⋅⋅(H0+δ1HE+{∑i≠EN33i−∑i≠EN22i+N33E−N22E}M0)

Let us subtract Equation (32) from Equation (33), retaining in the resulting equation only terms of the first order of smallness in *^1^δH_E_*, *N_11_^E^*, *N_22_^E^*, *N_33_^E^*:

(34) M0Q1(N11E−N22E)+M0Q3(N33E−N22E)+Q2δ1HE=0

where:

(35) $$\begin{array}{l} Q_{1}=H_{0}+\left\{\sum_{i\neq E}N_{33}^{i}-\sum_{i\neq E}N_{22}^{i}\right\}M_{0}\\ Q_{2}=2H_{0}+\left\{\sum_{i\neq E}N_{11}^{i}+\sum_{i\neq E}N_{33}^{i}-2\sum_{i\neq E}N_{22}^{i}\right\}M_{0}\\ Q_{3}=H_{0}+\left\{\sum_{i\neq E}N_{11}^{i}-\sum_{i\neq E}N_{22}^{i}\right\}M_{0} \end{array}$$

Now we find the resonance line shift:

(36) δ1HE=M0[Q1(N22E−N11E)+Q3(N22E−N33E)]Q2

Substitute Equations (27) and (28) into Equation (35):

(37) $$\begin{array}{l} Q_{1}=H_{0}-H_{a}+M_{0}\\ Q_{2}=2\left(H_{0}-H_{a}\right)+M_{0}\\ Q_{3}=H_{0}-H_{a} \end{array}$$

Furthermore, from Equation (36) we find:

(38) δ1HE=3λ100(Q1{Tm1−Tm2}−Q3Tm2)μ0M0Q2

The case when the bias field is directed perpendicular to the YIG plate is considered similarly. It turns out to be the following equation:

(39) δ2HE=3λ100[Tm1+Tm2]2μ0M0

The material parameters used in the calculation are given in the previous section in Table 2. Ferrite and piezoelectric thicknesses are *^m^t* = 2 · 10^−5^ m, *^p^t* = 5 · 10^−4^ m, respectively. 

The material parameters of YIG (001) [3] and Langatate X-cut [34] are given in Table 3.

**Figure 7:**
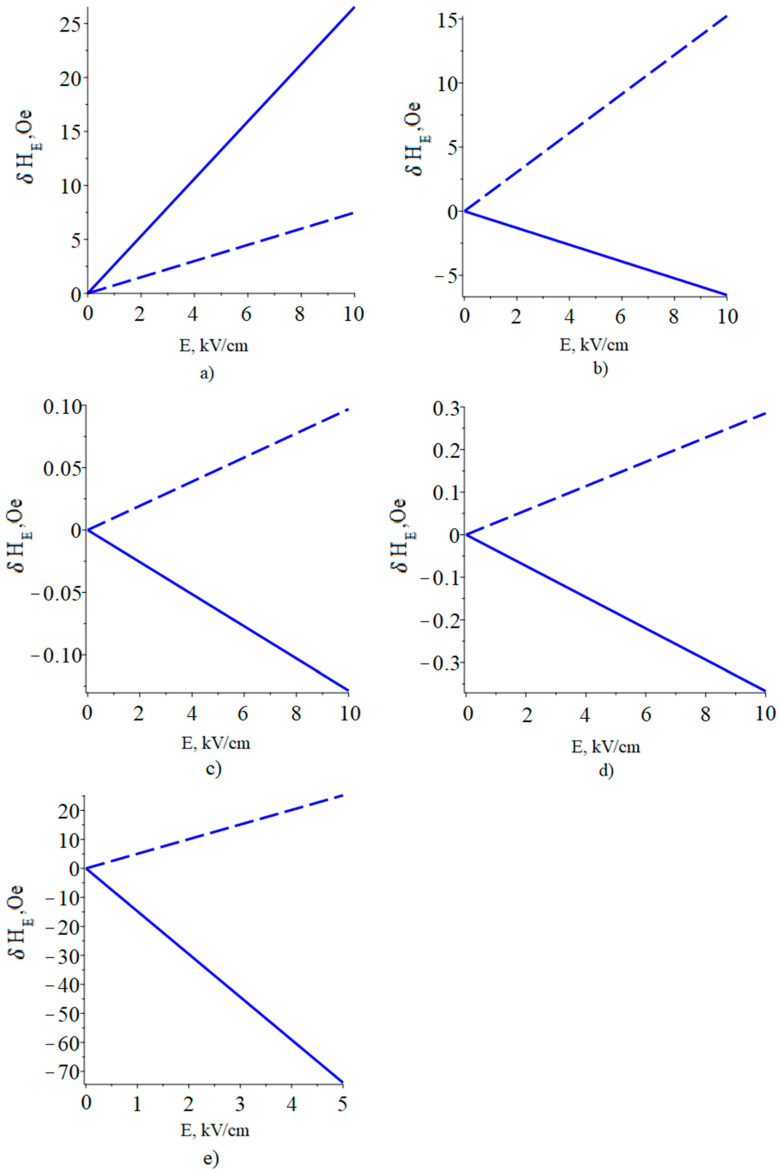
Dependence of the FMR line shift on the electric field in the structures: (**a**) YIG/PMN-PT, (**b**) YIG/PZT, (**c**) YIG/Quartz, (**d**) YIG/Langatate, (**e**) YIG/PZN-PT. Solid line is the bias field directed in the plane of the plate YIG along axis 2 (y), dash line is the bias field is directed perpendicular to the YIG plate along axis 3 (z).

The results of calculating the dependence of the FMR line shift on the electric field in YIG/piezoelectric structures are shown in Figure 7.

The largest FMR line shift was calculated for the YIG/PZN-PT ME composite, with a bias field oriented along the 2 (y) axis and in an electric field two times smaller than for the other ME composites.

Furthermore, high values of the FMR line shift were calculated for the YIG/PMN-PT ME composite with a bias field oriented along the 2 (y) axis.

This is due both to the fact that PZN-PT and PMN-PT have high piezoelectric coefficients and to the fact that the side edges of the (011) crystals of PMN-PT and PZN-PT coincide with the [100] and [01−1] crystallographic directions. In this case, the value of (*d_32_*-*d_31_*), which is proportional to the FMR line shift, has a maximum value. The decrease in the FMR line shift for PZT is because its piezoelectric coefficients are smaller than those of PMN-PT. In addition, due to the symmetry of the PZT, its values d_31_ and d_32_ coincide. Since the piezoelectric coefficients of quartz and langatate are approximately two orders of magnitude lower than those of PZT and PMN-PT, the FMR line shift in an electric field is relatively small for these materials.

As a result, since the width of the FMR line for YIG is about 1 Oe, it seems possible to experimentally observe the FMR line shift in an electric field in the considered ME structures.

## 4. ME Effect in Piezoelectric –YIG–GGG–“Substrate Effect”

**Figure 8:**
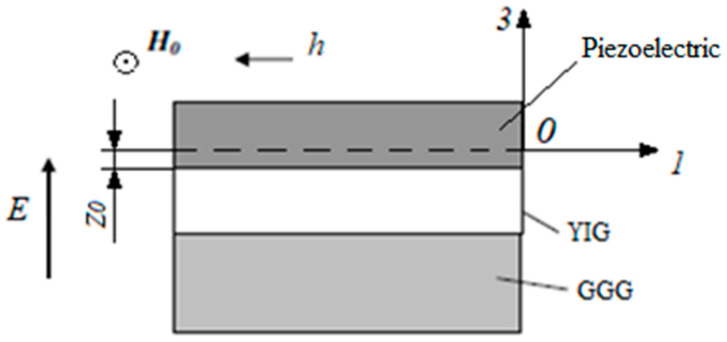
Structure of the ME composite PMN-PT/YIG/GGG.

In practice, a thin epitaxial layer of the YIG magnetostrictive ferrite phase is formed on a GGG substrate. In this paragraph, we consider in our theoretical model the effect of the GGG substrate on the FMR line shift for ME piezoelectric/YIG/GGG (Figure 8) structures and compare these results with those obtained earlier, when the need for the substrate was not included.

Let us consider a thin YIG film to which a bias field *H_0_* is applied along the plane of the YIG film. We will assume that the value of this field is sufficiently large and the thin YIG film is uniformly magnetized to saturation. Axis 2 (y) is directed along *H_0_*, then *H_0_* has components (0, H_0_, 0), and the equilibrium magnetization has components (0, M_0_, 0). Let us direct the axis 1 (x) along the edge of the GGG plate, which coincides with the crystallographic direction of the YIG [100].

The values of the bias field *H_0_* for a thin YIG film for two orientations of the bias field are the same as in the previous section.

Let us find the demagnetizing factors for the PMN-PT/YIG/GGG structure associated with the action of a constant electric field. Let us consider the case when the constant electric field is directed along the 3 (z) axis. Since both longitudinal and bending modes are excited along both X and Y axes in an asymmetric ME structure, the components of the strain tensor along axes 1 and 2 have the form:

(40) $$S_{1}=A_{1}+zB_{1}$$

(41) $$S_{2}=A_{2}+zB_{2}$$

where *A_1_*, *A_2_* and *B_1_*, *B_2_* are unknown constants associated with the planar and bending modes, respectively.

Consider the mechanical equilibrium of the structure. Boundary conditions for a free sample in the form of a rectangular parallelepiped:

Component stress tensors of the ME composite along axis 3 have the form:

(42) Ts3=Tm3=Tp3=0

for longitudinal forces in the ME composite along axes 1 and 2:

(43) $$\begin{array}{l} F_{1}=0\\ F_{2}=0 \end{array}$$

for bending moments in the ME composite along axes 1 and 2:

(44) $$\begin{array}{l} M_{1}=0\\ M_{2}=0 \end{array}$$

The constitutive Equations (13) and (14) hold for YIG and for piezoelectric, respectively.

Taking into account Equation (42), the constitutive equations for substrate:

(45) S1=ss11Ts1+ss12Ts2S2=ss12Ts1+ss11Ts2

From Equations (13), (14) and (45) we express the necessary stress components:

(46) Tm1=cm11S1+cm12S2Tm2=cm11S2+cm12S1Tp1=cp11ES1+cp12ES2+h31ETp2=cp22ES2+cp12ES1+h32ETs1=cs11S1+cs12S2Ts2=cs11S2+cs12S1

where cm11, cm12; cp11E, cp12E, cp22E, cs11, cs12 are the effective stiffness coefficients of the magnetic, piezoelectric and substrate, respectively; *h_31_*, *h_32_* are piezo coefficients.

We determine the longitudinal forces as:

(47) F1=∫−z0−tm−ts−z0−tmTs1dz+∫−z0−tm−z0Tm1dz+∫−z0tp−z0Tp1dzF2=∫−z0−tm−ts−z0−tmTs2dz+∫−z0−tm−z0Tm2dz+∫−z0tp−z0Tp2dz

and the bending moments as:

(48) M1=∫−z0−tm−ts−z0−tmzTs1dz+∫−z0−tm−z0zTm1dz+∫−z0tp−z0zTp1dzM2=∫−z0−tm−ts−z0−tmzTs2dz+∫−z0−tm−z0zTm2dz+∫−z0tp−z0zTp2dz

Substituting the necessary components of the stresses and strain tensors and integrating in Equations (47) and (48), we group the resulting factors in front of the unknown constants *A_1_*, *A_2_*, *B_1_*, *B_2_*, and the electric field *E*. Furthermore, we substitute the obtained longitudinal forces and bending moments into Equations (43) and (44):

(49) $$\begin{array}{l} F_{1}=-A_{1}J_{1}-A_{2}J_{2}+B_{1}P_{1}+B_{2}P_{2}-EY_{3}=0\\ F_{2}=-A_{1}J_{2}-A_{2}J_{1}+B_{2}P_{1}+B_{1}P_{2}-EY_{4}=0\\ M_{1}=A_{1}P_{1}+A_{2}P_{2}+B_{1}D_{1}+B_{2}D_{2}-EY_{1}=0\\ M_{2}=A_{1}P_{2}+A_{2}P_{1}+B_{2}D_{1}+B_{1}D_{2}-EY_{2}=0 \end{array}$$

where:

(50) D1=−cs11[(z0+tm+ts)3−(z0+tm)3]3+cm11[z03−(z0+tm)3]3−cp11E[z03+(tp−z0)3]3D2=−cs12[(z0+tm+ts)3−(z0+tm)3]3+cm12[z03−(z0+tm)3]3−cp12E[z03+(tp−z0)3]3P1=cs11[(−z0−tm−ts)2−(−z0−tm)2]2+cm11[(−z0−tm)2−z02]2+cp11E[z02−(tp−z0)2]2P2=cs12[(−z0−tm−ts)2−(−z0−tm)2]2+cm12[(−z0−tm)2−z02]2+cp12E[z02−(tp−z0)2]2Y1=h31(z02−(tp−z0)2)2Y2=h32(z02−(tp−z0)2)2Y3=h31tpY4=h32tpJ1=cs11ts+cm11tm+cp11EtpJ2=cs12ts+cm12tm+cp12Etp

To eliminate the dependence of the longitudinal forces along the axes 1 and 2 on the bending deformations along the axes 2 and 1, respectively, we equate to zero the multiplier *P_2_* in front of the unknown constant *B_2_* for the longitudinal force *F_1_*, which is also the multiplier in front of the unknown constants *A_1_*, *A_2_*, *B_1_* for *M_2_*, *M_1_* and for *F_2_*, respectively.

(51) cs12[(−z0−tm−ts)2−(−z0−tm)2]2+cm12[(−z0−tm)2−z02]2+cp12E[z02−(tp−z0)2]2=0

However, even now, the solution of the system of 4 linear inhomogeneous equations for 4 unknowns *A_1_*, *A_2_*, *B_1_*, *B_2_* (49), looks cumbersome. Therefore, we do not present it here but use it only in further calculations to obtain the final result. From Equation (51), we find the distance from the neutral line of the composite to the interface between the YIG and the piezoelectric:

(52) z0=tp2cp12E−tm2cm12−tscs12(2tm+ts)2(tpcp12E+tmcm12+tscs12)

Since the stress components of the magnetostrictive phase will depend on *z*, but the thickness of the magnetostrictive phase is small, then we can take for *z* the coordinate of this phase middle as:

(53) z=−tm2−z0

We substitute the found constants *A_1_*, *A_2_*, *B_1_*, *B_2_* into Equations (40) and (41), and then we substitute the obtained strain tensor components along axes 1 and 2 into the YIG stress components *^m^T_1_*, *^m^T_2_* from Equation (46).

**Figure 9:**
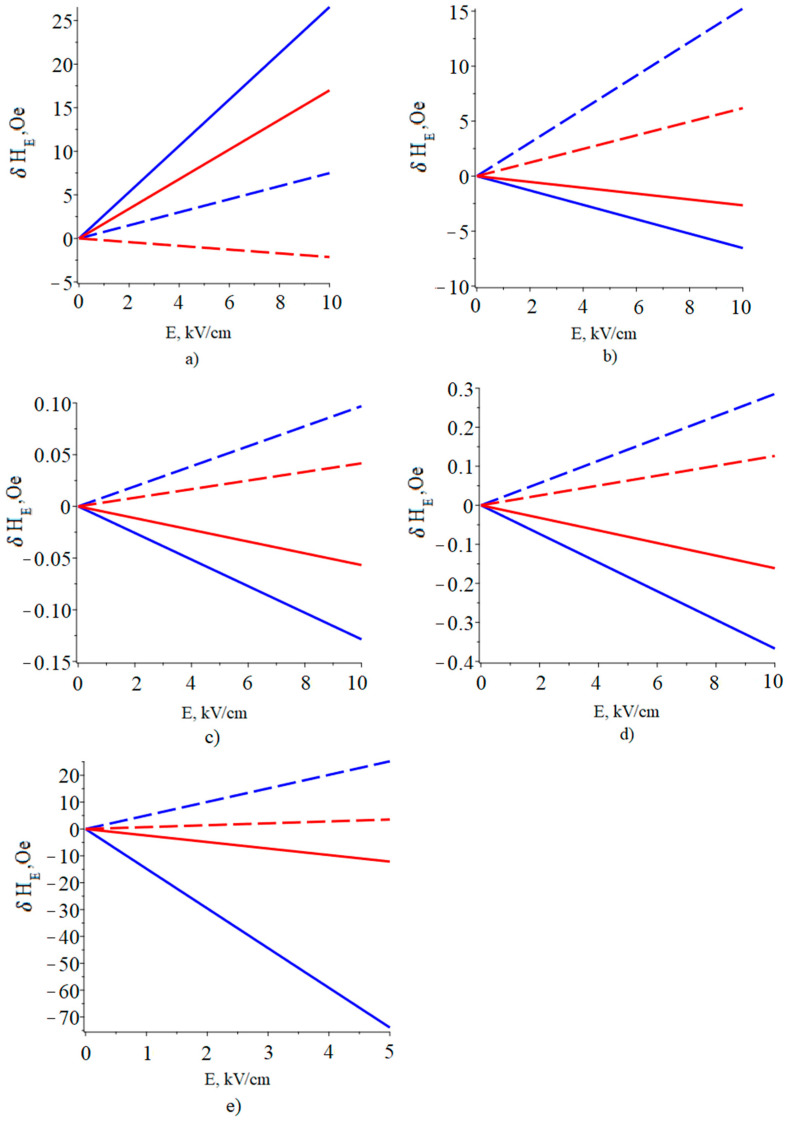
Dependence of the FMR line shift on the electric field in the structures: (**a**) YIG/PMN-PT is blue line, PMN-PT/YIG/GGG is red line, (**b**) YIG/PZT is blue line, PZT/YIG/GGG is red line, (**c**) YIG/Quartz is blue line, Quartz/YIG/GGG is red line, (**d**) YIG/Langatate is blue line, Langatate/YIG/GGG is red line, (**e**) YIG/PZN-PT is blue line, PZN-PT/YIG/GGG is red line. Solid line is a bias field directed in the plane of the plate YIG along axis 2 (y), dash line is a bias field is directed perpendicular to the YIG plate along axis 3 (z).

Furthermore, the YIG stress components found in this way (*^m^T_1_*, *^m^T_2_*), in which the influence of the substrate and bending vibrations are now taken into account, are substituted into Equation (38), which gives the FMR line shift at a bias field directed along the plane of the YIG thin film. Similarly, to calculate the FMR line shift for the case when the bias field is directed perpendicular to the thin film, the found YIG stress components *^m^T_1_*, *^m^T_2_* are substituted into Equation (39). These calculations were used to plot the dependence of the FMR line shift on the magnitude of the applied electric field to the ME composite of piezoelectric/YIG/GGG for two cases of the bias field orientation: a bias field directed in the plane of the plate YIG along axis 2 (y) and perpendicular to the YIG plate along axis 3 (z). For ease of comparison, in Figure 9 the corresponding graphs for the ME composite of piezoelectric/YIG are shown.

Parameters of GGG: *^s^s_11_* = 4.4 · 10^−12^ m^2^/N,^*s*^*s_12_* = −1.2 · 10^−12^ m^2^/N, *t* = 5 · 10^−4^ m.

Below, the graphs of the dependence of the FMR line shift on the applied electric field to the ME composite of piezoelectric/YIG/GGG are shown.

Based on the results of the calculations, it was determined that for the piezoelectric/YIG/GGG structures, the FMR line shift is approximately two times less than for the YIG/piezoelectric structures for both types of orientation of the bias field. For ME composite PZN-PT/YIG/GGG, the FMR line shift is approximately seven times less than for ME composite YIG/PZN-PT. Furthermore, for the PMN-PT/YIG structure, taking into account the GGG substrate, a change in the sign of the FMR line shift for a bias field that is perpendicular to the YIG plate is observed. The results of the calculation confirm the “clamping effect” that occurs in the presence of a substrate. This effect is due to the fact that mechanical deformations that occur in a piezoelectric under the action of an electric field in the presence of a substrate are transferred not only to the magnetostrictive phase but also to the substrate. This weakens the mechanical stresses arising in the YIG, compared with the case without the substrate, and leads to a decrease in the FMR line shift. It should be noted that bending strains differ from planar strains in that the strain tensor components above and below the neutral plane have different signs. Therefore, when considering the substrate in our model, we simultaneously included the bending deformations for the PMN-PT / YIG / GGG structures, and the sign of the FMR line shift changed compared with the PMN-PT / YIG structures.

## 5. Discussion

It is of interest to discuss several issues related to the prospect of increasing the efficiency of the microwave ME effect and ME devices. In many cases, to observe the microwave ME effect, it is necessary to apply a rather strong bias field to the magnetostrictive phase. To create it, either permanent magnets or an additional electromagnetic coil are required, which leads to an increase in the mass and dimensions of ME devices. To reduce the weight and size parameters, it is necessary to achieve a decrease in the value of the required bias field. One option is to use internal fields of uniaxial magnetic anisotropy. In ref. [35], the authors, using this method, observed the microwave ME effect at zero bias field at the FMR resonant frequency of 1.75 GHz. Another way to create the necessary internal bias field without the use of external magnetic field sources is to use a gradient magnetostrictive self-biased phase [3]. In particular, two layers of different magnetic materials can be used, one of which is magnetically hard and the other is magnetically soft. By choosing the required ratio of layer thicknesses, it is possible to create the necessary internal bias field. Furthermore, one of the important issues is reducing the magnitude of the control electric field. One way to solve this problem is to use new piezoelectric materials with increased piezoelectric coefficients. As shown in the article, the use of PZN-PT and PMN-PT already allows, with the same magnitude of the microwave ME effect, to significantly reduce the magnitude of the control electric field compared with other piezoelectrics. The performed mathematical modeling has shown that the use of a passive substrate in the ME structure significantly reduces the magnitude of the microwave ME effect. To preserve the effect, it is necessary either to weaken the influence of the substrate on the microwave ME effect or to find a way to dispense with the substrate. To weaken the influence of the substrate, if possible, reduce the thickness of the substrate and make it from a less mechanically rigid material or use a piezoelectric instead of the substrate [35]. Another negative factor affecting the magnitude of the microwave ME effect is the “mismatch effect”, i.e., the discrepancy between the parameters of the crystal lattices of the magnet, piezoelectric, and substrate. The influence of this factor can be estimated using the phenomenological thermodynamic theory of Landau–Ginzburg–Devonshire [36]. In order to avoid a mismatch of the crystal lattice parameters, it is recommended to use such cuts so that the crystal lattice parameters differ as little as possible from each other at the interface.

## 6. Conclusions

In the development of our previous calculations, here we considered the ME effect in the FMR region for FM-piezoelectric structures. Ni, Fe, Co, NiFe, and FeGaB were used as the FM, and Quartz, PZT, PMN-PT, and PZN-PT were used as the piezoelectrics. A comparison of the obtained results shows that the maximum FMR line shift is observed for Fe, and the minimum for NiFe in structures with the PZN-PT piezoelectric and is of 450 Oe and of 25 Oe, respectively, in an electric field of 5 kV/cm. When PZN-PT is replaced by Quartz, the ME effect sharply decreases by 3 orders of magnitude for the Fe- structures. These differences are due to the various values of the material parameters of the FM and piezoelectrics in accordance with Equation (25). Based on the results obtained, a simple practical conclusion can be drawn: to obtain a large FMR line shift, it is necessary to use piezoelectrics with a high permittivity. In contrast to the previous results, the calculation of the ME effect in the YIG-piezoelectrics and YIG-GGG-piezoelectric structures made it possible to analyze the “substrate effect” and evaluate the strain-mediated microwave ME effect. In the case of YIG-based structures with the piezoelectrics, such as Quartz, langatate, PZT, PMN-PT and PZN-PT, by analogy with the previous study, we obtained the expected maximum shift for PZN-PT, and the minimum for Quartz, which was of 70 Oe and of 0. 05 Oe, respectively. A detailed calculation of the “substrate effect” in the electric field in the considered structures quantitatively confirmed its significant decrease, in particular, for the piezoelectric-YIG-GGG structures, as compared with the piezoelectric-YIG structures, where the FMR line shift decreased on average by a factor of 2, which is associated with the redistribution of strain between the YIG layer and substrate. 

An analysis of the studies carried out and the results obtained allow us to determine the future directions, which should include the following:

(a) It is necessary to carry out coordinated theoretical and experimental studies of the “substrate effect”, which is determined by the influence of the substrate on the magnitude of the ME effect, and the “mismatch effect”, which consists of considering the discrepancy between the parameters of the crystal lattices of the magnet, piezoelectric, and substrate.

For this purpose, special structures should be chosen so that the purposeful calculations could be compared with experimentally obtained data. In addition, one should keep in mind the possibility of depositing magnetic micro- and nanofilms directly onto a piezoelectric layer, which can play the role of a substrate [35]; 

(b) Of great interest is the experimental study of the ME effect in the region of shear and torsional modes in the EMR range. Since these modes are of higher frequency, it becomes possible to proceed to the study of the ME effect in the region of MAR [3]. Preliminary estimates show the possibility of obtaining giant ME effects in the MAR region, but the difficulty of observing this effect lies in the need to match the frequency of one of the EMR modes with the FMR frequency in the structure under study. Since the frequency ranges of EMR and FMR differ significantly, it seems that to observe this effect, the FMR frequency should be lowered by choosing a magnetic component with a low saturation magnetization, and the EMR frequency should be increased by reducing the thickness of the piezoelectric layer and using EMR harmonics; 

(c) To improve the efficiency of the inverse microwave ME effect, it is necessary to use magnetic films with a narrow magnetic resonance line (FMR linewidth less than 10 Oe), low values of the bias field, and a control electric field. The production of magnetic films with narrow resonance lines is a serious technological problem that must be solved in the future. An example in this regard is a YIG film on a GGG substrate, which has an FMR linewidth of less than 1 Oe. Since the magnitude of the bias field is related to the demagnetizing factors and the saturation magnetization, it is necessary to choose the tangential orientation of the bias field and the magnetic film with the minimum saturation magnetization. As for the control voltage, the estimates show that an electric voltage of 1 V can provide an FMR line shift of several line widths for a piezoelectric film thickness of up to 1 μm [26]; 

(d) It is known that for a number of orientations of the bias field different from the main crystallographic directions, the values of the shift and broadening of the FMR lines significantly exceed the values calculated for the strain-mediated ME effect. To adequately explain this effect, it is necessary to carry out experimental studies on various ME structures involving detailed calculations of the two-magnon scattering and surface charge [9,10,11,12,13].

## Figures and Tables

**Table 1:** Material parameters of ferromagnetic metals and alloys [3].

Material	Ni	Fe	Co	NiFe	FeGaB
s_11_ (10^−12^ m^2^/N)	20	5	4.7	6.67	18.2
s_12_ (10^−12^ m^2^/N)	−7	−1.45	−2.3	−1.93	−4
M_0_ (A/m)	262,698	101,587	1,153,968	1,230,158	1,110,000
γ (m/C)	236,803	228,910	236,803	220,000	221,017
λ_100_ (10^−6^)	−35	−8	−50	4.6	70
H_a_ (A/m)	15,900	15,662	1401	0	1990

**Table 2:** Material parameters of piezoelectrics.

Material	Cut (011) of PZN-PT	Cut (011) of 0.67PMN-0.33PT (PMN-PT)	PZT	X-cut of Quartz
s_11_ (10^−12^ m^2^/N)	54	69	15.3	12.8
s_12_ (10^−12^ m^2^/N)	−41.6	−33.4	−5	−1.22
s_22_ (10^−12^ m^2^/N)	180	22.9	15.3	9.6
d_31_ (10^−12^ m/V)	1100	−940	−175	0
d_32_ (10^−12^ m/V)	−3000	475	−175	−2.29

**Table 3:** Material parameters of YIG (001) [3] and Langatate X-cut [34].

Material	YIG (001)	Langatate X-Cut
s_11_ (10^−12^ m^2^/N)	4.8	5.27
s_12_ (10^−12^ m^2^/N)	−1.4	−1.84
s_22_ (10^−12^ m^2^/N)	-	9.13
M_0_ (A/m)	142,143	-
λ_100_ (10^−6^)	−1.4	-
H_a_ (A/m)	−3343	-
d_31_ (10^−12^ m/V)	-	0
d_32_ (10^−12^ m/V)	-	−6.54

## Data Availability

Not applicable.
